# Bioconvection flow in accelerated couple stress nanoparticles with activation energy: bio-fuel applications

**DOI:** 10.1038/s41598-021-82209-0

**Published:** 2021-02-08

**Authors:** Sami Ullah Khan, Kamel Al-Khaled, A. Aldabesh, Muhammad Awais, Iskander Tlili

**Affiliations:** 1 Department of Mathematics, COMSATS University Islamabad, Sahiwal, 57000 Pakistan; 2 Department of Mathematics and Statistics, Jordan University of Science and Technology, P.O. Box 3030, Irbid, 22110 Jordan; 3 Department of Mechanical Engineering, Faculty of Engineering, Albaha University, Al Bahah, 65527 Saudi Arabia; 4 University Institute of Biochemistry and Biotechnology, PMAS-Arid Agriculture University, Rawalpindi, 43600 Pakistan; 5 Institute of Research and Development, Duy Tan University, Da Nang, 550000 Vietnam; 6 Faculty of Civil Engineering, Duy Tan University, Da Nang, 550000 Vietnam

**Keywords:** Fossil fuels, Mechanical engineering

## Abstract

On the account of significance of bioconvection in biotechnology and several biological systems, valuable contributions have been performed by scientists in current decade. In current framework, a theoretical bioconvection model is constituted to examine the analyzed the thermally developed magnetized couple stress nanoparticles flow by involving narrative flow characteristics namely activation energy, chemical reaction and radiation features. The accelerated flow is organized on the periodically porous stretched configuration. The heat performances are evaluated via famous Buongiorno’s model which successfully reflects the important features of thermophoretic and Brownian motion. The composed fluid model is based on the governing equations of momentum, energy, nanoparticles concentration and motile microorganisms. The dimensionless problem has been solved analytically via homotopic procedure where the convergence of results is carefully examined. The interesting graphical description for the distribution of velocity, heat transfer of nanoparticles, concentration pattern and gyrotactic microorganism significance are presented with relevant physical significance. The variation in wall shear stress is also graphically underlined which shows an interesting periodic oscillation near the flow domain. The numerical interpretation for examining the heat mass and motile density transfer rate is presented in tubular form.

## Introduction

Owing to the convinced thermal importance of nanoparticles and their novel importance in industries, bio-medical and engineering sciences, much focus is elaborated by dynamic scientists in current century. Nanoparticles are mixture of small sized metallic particles (1–100 nm) with enhanced thermo-physical properties. Nanoparticles obey a number of interrelationships that may contribute to creation of a specific turbidity patterns or density delimitation, formation of nanoparticles and buoyant forces. The nanofluids have been reported to have more thermal conductivity values as compared with other ordinary fluids. The basic idea of such metallic nanoparticles was conveyed by Choi^1^. The experimental based investigation presented by Choi^1^ showed that low performances of traditional base liquids can by enhanced up to extraordinary level with utilization of such nanoparticles. Later on, Buongiorno^2^ developed a non-homogeneous equilibrium model to explain the slip mechanism of nanoparticles by introducing Brownian movement and thermophoresis features. Owing to higher thermal conductivity content of nanofluids and optical activity values, these fluids are also having applications in the fields of power and heat generations, biotechnology, light based chemical sensor, nano-medicines and cooling systems. Recently many numerous investigators reported their analysis with utilization of such nanoparticles. For instance, Sheikholeslami and Bhatti^3^ spotted that change of shape and velocity of nanofluid is increased with increasing Reynold and Darcy numbers and the platelet shape was reported the best for maximum heat transfer. Babu and Sandeep^4^ discussed nano-material properties subject to the slip mechanism confined by a slandering surface. They employed the Buongiorno’s nanofluid model to search out the Brownian and thermophoretic assessment. Hamid et al.^5^ reported rotating flow MoS_2_ nanoparticles with shape effects and variable thermal conductivity. Imtiaz et al.^6^ claimed that single wall carbon nanotubes (SWCNTs) are potentially more feasible for less molecular drag and more heat transfer as compared with multiwall carbon nanotubes (MWCNTs). The nanoparticles utilization for stretched flow of Jeffrey material was directed by Khan and co-investigators^7^. The comparative studies conducted by Mahanthesh et al.^8^ deals with the flow nanofluid over a stretched surface in presence of Hall effects and heat source/sink features. The effects of injection and suction in flow of iron based nanoparticles were validated by Abuzar and Turkyilmazoglu^9^. Their results showed that presence of injection and suction offer resistance to the settlement of nanoparticles. The study performed by Khan et al.^10^ revealed that thermophoresis and Brownian factors effectively enhanced the nanoparticles temperature configured by a periodically accelerated surface. Aly^11^ computed interesting dual solution for interpolating the thermal efficiency of graphene nanoparticles in presence of suction phenomenon. Khan and co-investigators thrashed out the thermal characteristics of nanoparticles configured by an accelerated surface and showed that that variation in Hartmann number and thermophoresis parameters enhanced the nanoparticles temperature. The study of water-based nanoparticles with interaction of magnetic field has been numerically worked out by Tlili et al.^13^. Uddin et al.^14^ examine thermal radiation and slip mechanisms in convective flow of nanofluid organized over a moving configuration. In another useful contribution, Uddin and co-workers^15^ utilized multiple slip features in bio-convection flow of nanofluid confined by horizontal plate with help of numerical algorithm. The slip flow in a horizontal channel filled by nano-materials subject to magnetic forces was reported by Zohra et al.^16^. Beg and co-workers^17^ involved Stefan blowing and Navier slip consequences to examine the heat and mass transfer aspects od nanofluid configured by rotating cone. Uddin et al.^18^ employed the Chebyshev collocation numerical scheme to investigate the flow of nanofluid in presence of magnetic induction and multiple slip features. The study of bio-nano-convective materials additionally impacted by magnetic force and Stefan blowing effects confined by rotating disc has been analyzed by Zohra et al.^19^.

Modern day investigations on bioconvection mainly focus on the increasing heat and mass transfer with applications in chemical, mechanical, process, civil and electronics engineering. More specifically, the cooling systems required for electronic instruments, building insulations along with geothermal nuclear waste disposal are the advanced bioconvection application sectors. Furthermore, the micro-channels thermal sinks, micro-heat pipes and micro-reactors have further extended the span of current bioconvection research. In biological and biotechnological systems the bioconvection applications for blood flow, biosensors, micro-enzyme, nano-biotechnology and biomedical instrumentation studies have made it possible to study drug delivery, pharmacokinetics, content detection and nano-medicine with more details. The convection (molecular heat transfer) is referred to the transportation of heat transfer inside the matter. Naturally it can be observed with hot and cold winds that bring change in the environmental temperatures. Similarly, microorganisms are also considered as way of convection in materials which represent a mimic the motion of particles in nano fluids microorganisms. Unicellular microbes move randomly in a colloidal solution and distribute themselves differentially within a suspension creating a density stratification. This density stratification is usually brought up by the “Bioconvection” phenomenon which is spontaneous movement of individual microbial cells according to the relative colonial densities. The spontaneous microbial movements are mostly brought about by the specific stimuli that affect the randomized cellular distribution in a colloid. Therefore,microbial cells move in response to light, chemicals, oxygen, density and gravity etc. the movement of microbes according to the above mentioned stimuli is referred as phototaxis, chemotaxis, oxytaxis, gyrotaxis and gravitaxis etc. Most of the microbes show enormous sensitivity to the stimulus of light therefore their movements can be controlled by using electromagnetic waves. Consequently, the bioconvection patterns in a colloidal suspension with phototactic microorganisms can be controlled by illuminations. Moreover, other stimuli can also be exploited to demonstrate differential bioconvection patterns using chemotactic or other microbial cultures. Firstly, the bioconvection can also be helpful in biotechnology for biofuels. The algal biofuel systems where algae are artificially incubated or the biofuel reactors where anaerobic digestion is a key to biofuel production the movement of microbes is a main concern. The movement of microbial community can be artificially varied and optimized to gain a specific light penetration, distribution of biomass and finally the higher surface area for microbes to digest biomass. The photo-gyrotactic movements can help in establishing optimal turbulent stirring during algal growth and steady state light penetration with increasing depth of algal growth cultures. Secondly, biosensors development is another field where Bioconvection has a potential that can create a quantitative and qualitative detection gateway for biomolecules. Furthermore, a biomimetic system was also developed to evaluate the inflammatory and toxic responses posed by nano allergens. Acknowledging to the fundamental continuation regarding bioconvection pattern of nanoparticles in presence of motile microorganisms has been directed by Kuznetsov^20,21^. The effect of mass and thermal slip for nanofluid significance with mixed convection applications was numerically evaluated by Uddin et al.^22^. Moreover, the heat, mass transfer effects with utilization of gyrotactic microorganism over a cone were presented by Siddiqa and co-workers^23^. Uddin et al.^24^ explained the bioconvection across wavy surfaces and established that local motile density number get decrement trend with evaluation of Peclet constant. The study conducted by Farooq et al.^25^ deals with the mixed convection slip flow of Sisko nanoparticles along with gyrotactic microorganism. The heat and mass transfers are the key parameters associated with bioconvection bearing wide variety of applications. The bio-convective transportation concern the mass and heat transfer assessment in flow for time dependent flow of nanoparticles has been worked out by Waqas et al.^26^. The bio-convective patterns around a truncated cone were also observed by Khan and co-researchers^27^ and elucidated bioconvection flow of nano-material over truncated cone. The investigation for bioconvection flow of nanofluid in presence of Stefan blowing features has been suggested by Amirsom et al.^28^. Dero et al.^29^ evaluated the slip effects and Stefan blowing effects in flow of nanofluid configured by shrinking surface. Kiari et al.^30^ investigated the some novel thermal consequences in viscous dissipation, chemical reaction and thermal radiation in bio-convective flow of Casson nanofluid subject to the suction and injection applications. Shaw et al.^31^ presented the applications of oxytactic micro-organisms in non-Darcy porous space confined by a horizontal plate. The multiple slip effects in bio-convective analysis of non-Newtonian fluid have been numerically reported by Nayak et al.^32^. The investigation claimed by Shaw et al.^33^ claimed the bioconvection aspects in order to analyze the thermal assessment of water-based nanofluid in a porous space. In another investigation Shaw et al.^34^ utilized the soret features in magnetized flow of nanofluid in presence of gyrotactic microorganisms. Magagula et al.^35^ inspected the double dispersed flow of Casson nano-material containing gyrotactic microorganisms with additional features of first order chemical reaction and nonlinear thermal radiation.

Due to immense industrial and significance of non-Newtonian material, a supreme interest has been developed by scientists to examine the complex rheology of such non-Newtonian fluids. The interesting applications of such complex materials includes wire coating, food manufacturing, oils, greases, blood, petroleum industries etc. To confirm the discriminative aspects of non-Newtonian fluid materials, these materials are characterized into various models based on complex constitutive relations. Couple stress fluid is one which encountered size dependent features which cannot be explain by using classical viscous presumption. The speculation of couple stress is basically extension of traditional viscous mathematical model which encountered couple stress and body couples. The primly work on the rheology of couple stress fluid was proposed by Stokes^36^ which was further extended by many investigators^12,37–41^.

Inspired by above listed literature and applications, the central accent of current exploration is to investigate the bioconvection phenomenon of couple stress nanofluid with contains gyrotactic microorganisms over periodically accelerated surface. The nonlinear mathematical expression for radiative flux is captured in the heat equation. The activation energy applications are encountered in the concentration equation. The fundamental idea and work on flow induced by periodically accelerated geometry was induced by Wang^42^ and later on extensive investigations were led by numerous researchers^43–46^. Till now, no such study is reported in the literature with these flow features. The analytical solution is based on homotopic technique and variation of each flow parameter is graphically underlined.

## Problem description

**Figure 1:**
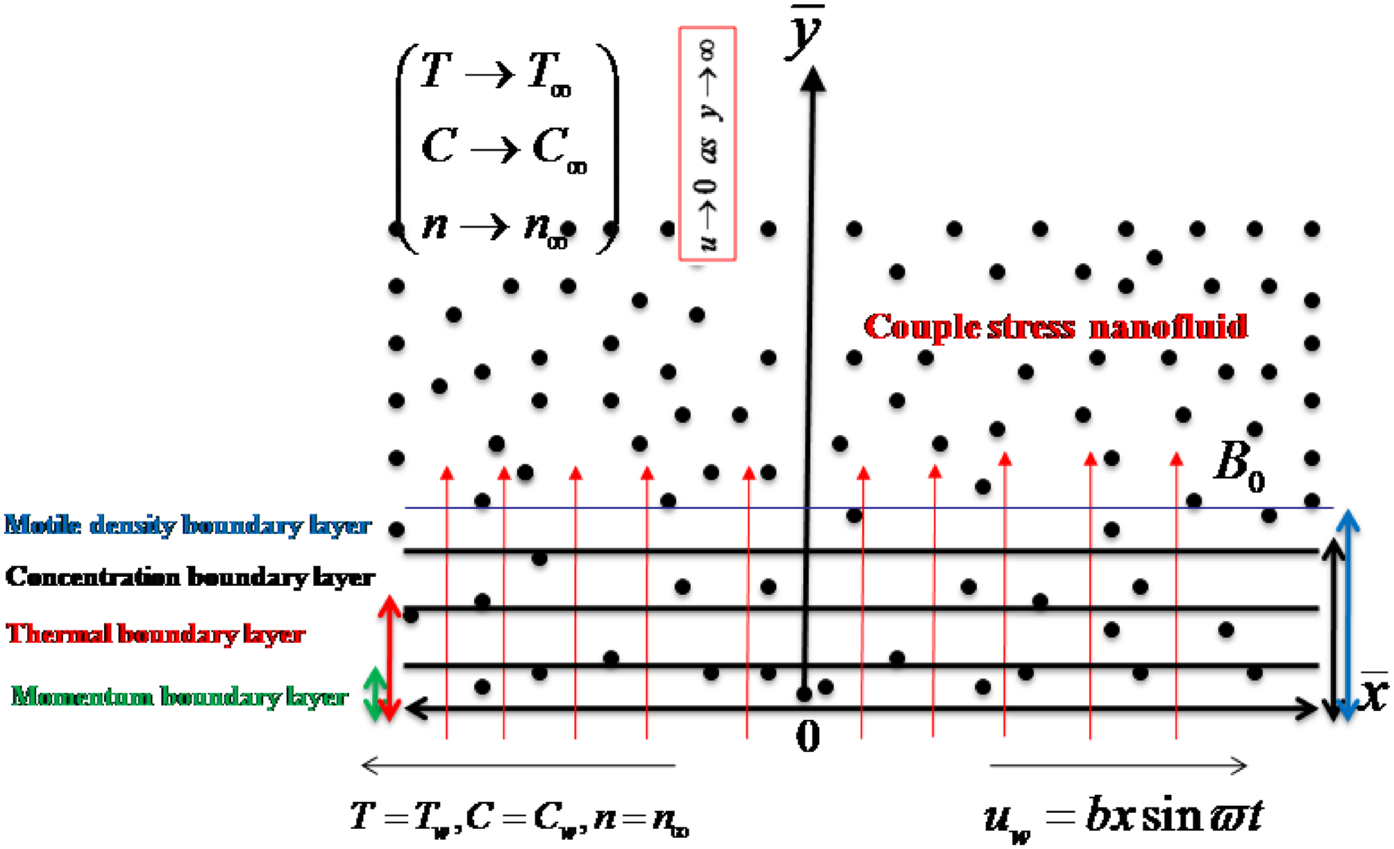
Geometry of flow problem.

We have assumed 2-D velocity pattern for couple stress nanofluid flow which is confined by an accelerated moving surface where magnetic field effects are utilized normally. Following to the coordinate system, $u$ (velocity component) has been taken in $x$-directions while $v$ is considered along $y$-axis. The stretched surface has been accelerated with uniform velocity $u = u_{\omega } = bx\sin \varpi t,$ where $\varpi$ being frequency and $b$ is stretching rate. The energy equation retained thermal radiation via theory of Rosseland approximations while activation energy expressions are entertained in equation of concentration. Let the convectively heated surface occupied the surface temperature $T_{w} ,$ surface concentration $C_{w}$ and motile microorganisms $N_{w} .$ Further, $T_{\infty } ,$ $C_{\infty }$ and $N_{\infty }$ are respectively free stream temperature, free stream concentration and free stream motile microorganisms as shown in Fig. 1. The magnetized couple stress fluid is subjected to a uniform magnetic force which is employed perpendicular to the accelerated and stretched surface. The utilization of these features, the developed governing representing the bioconvection flow of couple stress nanofluid are^12,38,50,51^:

1 $$\frac{\partial u}{{\partial x}} + \frac{\partial v}{{\partial y}} = 0,\,$$

2 $$\frac{\partial u}{{\partial t}} + u\frac{\partial u}{{\partial x}} + v\frac{\partial u}{{\partial y}} = \nu \frac{{\partial^{2} u}}{{\partial \overline{y}^{2} }} - \frac{{\eta_{0} }}{{\rho_{f} }}\frac{{\partial^{4} u}}{{\partial y^{4} }} - \frac{{\sigma_{e} B_{0}^{2} }}{{\rho_{f} }}u + \frac{1}{{\rho_{f} }}\left[ \begin{gathered} \left( {1 - C_{\infty } } \right)\rho_{f} \beta^{ * } g\left( {T - T_{\infty } } \right) - \left( {\rho_{p} - \rho_{f} } \right)g\left( {C - C_{\infty } } \right) \hfill \\ - \left( {n - n_{\infty } } \right)g\gamma^{ * } \left( {\rho_{m} - \rho_{f} } \right) \hfill \\ \end{gathered} \right],$$

3 $$\frac{\partial T}{{\partial t}} + u\frac{\partial T}{{\partial x}} + v\frac{\partial T}{{\partial y}} = \left( {\alpha_{1} + \frac{{16\sigma^{ * } T_{\infty }^{3} }}{{3k^{ * } \left( {\rho c} \right)_{f} }}} \right)\frac{{\partial^{2} T}}{{\partial y^{2} }} + \tau_{1} \left[ {D_{B} \frac{\partial C}{{\partial y}}\frac{\partial T}{{\partial y}} + \frac{{D_{T} }}{{T_{\infty } }}\left( {\frac{\partial T}{{\partial y}}} \right)^{2} } \right],$$

4 $$\frac{\partial C}{{\partial t}} + u\frac{\partial C}{{\partial x}} + v\frac{\partial C}{{\partial y}} = D_{B} \frac{{\partial^{2} C}}{{\partial y^{2} }} + \frac{{D_{T} }}{{T_{\infty } }}\frac{{\partial^{2} T}}{{\partial y^{2} }} - K_{r}^{2} \left( {C - C_{\infty } } \right)\left( {\frac{T}{{T_{\infty } }}} \right)^{n} \exp \left( {\frac{{ - E_{a} }}{\kappa T}} \right),\,$$

5 $$\frac{\partial n}{{\partial t}} + u\frac{\partial n}{{\partial x}} + v\frac{\partial n}{{\partial y}} + \frac{{b_{1} W_{e} }}{{\left( {C_{w} - C_{\infty } } \right)}}\left[ {\frac{\partial }{\partial y}\left( {n\frac{\partial C}{{\partial y}}} \right)} \right] = D_{m} \left( {\frac{{\partial^{2} n}}{{\partial y^{2} }}} \right),$$

The physical quantities appeared in the above equations are couple stress fluid constant $\eta_{0} ,$ electrical conductivity $\sigma_{e}$,gravity $g,$ nanoparticles density $\rho_{p} ,$ motile microorganism particles density $\rho_{m} ,$ fluid density $\rho_{f} ,$ temperature $T,$ thermal diffusivity $\alpha_{1}$, Stefan-Boltzmann constant $\sigma^{ * } ,$ mean absorption constant $k^{ * } ,$ diffusion constant $D_{B} ,$ concentration $C,$ reaction rate $K_{r} ,$ activation energy $E_{a} ,$ magnetic field strength $B_{0} ,$ Boltzmann constant $\kappa ,$ chemotaxis constant $b_{1}$ and swimming cells speed $W_{e} .$

The boundary assumptions for the current flow analysis are^12,38,50,51^:

6 $$u = u_{\omega } = bx\sin \varpi t,\,\; \, \frac{{\partial^{2} u}}{{\partial y^{2} }}{ = 0, }v = 0,\, \, T = T_{w} ,\, \, C = C_{w} {,}n = n_{w} {\text{ at}}\;\;\;y = 0,\, \, t > 0,\,$$

7 $$u \to 0,\, \, \frac{{\partial^{2} u}}{{\partial y^{2} }} \to 0, \, T \to T_{\infty } {, }C \to C_{\infty } {,}n \to n_{\infty } {\text{ at}}\;\;\;\;y \to \infty .$$

Before analyze the rheological features of various flow parameters from constituted flow equations, we need achieve the non-dimensional form of these equations by inserting following dimensionless quantities^12,38,50,51^:

8 $$u = bxf_{\xi } \left( {\xi ,\,\tau } \right),\, \, v = - \sqrt {\nu b} f\left( {\xi ,\,\tau } \right)\,,\,\,\,\xi = \sqrt {\frac{b}{\nu }} y,\, \, \tau = t\varpi ,$$

9 $$\theta \left( {\xi ,\tau } \right) = \frac{{T - T_{\infty } }}{{T_{w} - T_{\infty } }},\phi \left( {\xi ,\tau } \right) = \frac{{C - C_{\infty } }}{{C_{w} - C_{\infty } }},\chi \left( {\xi ,\tau } \right) = \frac{{n - n_{\infty } }}{{n_{w} - n_{\infty } }}.$$

The utilization of above suggested quantities in Eqs. (1–5) yield

10 $$f_{\xi \xi \xi } - Sf_{\xi \tau } - f_{y}^{2} + ff_{\xi \xi } - Mf_{\xi } - Kf_{\xi \xi \xi \xi \xi } + \lambda \left( {\theta - Nr\phi - Rb\chi } \right) = 0,$$

11 $$\left[ {1 + \frac{4}{3}Rd\left\{ {1 + \left( {\theta_{w} - 1} \right)\theta } \right\}^{3} } \right]\theta_{\eta \eta } + Rd\left[ {3\left( {\theta_{w} - 1} \right)\theta_{\eta }^{2} } \right]\left[ {1 + \left( {\theta_{w} - 1} \right)\theta } \right]^{2} + \Pr \left[ \begin{gathered} - S\phi_{\tau } + f\phi_{\eta } \hfill \\ + Nb\theta_{\eta } \phi_{\eta } + Nt\left( {\theta_{\eta } } \right)^{2} \hfill \\ \end{gathered} \right] = 0,$$

12 $$\phi_{yy} + \frac{Nt}{{Nb}}\theta_{\xi \xi } - S(\Pr Le)\phi_{\tau } + \Pr Lef\phi_{\xi } - \Pr Le\sigma \left( {1 + \delta \theta } \right)^{n} \exp \left( {\frac{ - E}{{\left( {1 + \delta \theta } \right)}}} \right)\phi = 0,$$

13 $$\chi_{\xi \xi } - S\left( {Lb} \right)\chi_{\tau } + Lb\chi_{\xi } - Pe\left[ {\phi_{\xi \xi } \left( {\chi + \sigma } \right) + \chi_{\xi } \phi_{\xi } } \right] = 0,$$

The boundary conditions in transformed form are:

14 $$f_{\xi } \left( {0,\tau } \right) = \sin \tau , \, f\left( {0,\tau } \right) = 0, \, f_{\xi \xi \xi } \left( {0,\tau } \right) = 0,\theta (0,\tau ) = 1,\phi (0,\tau ) = 1,\chi (0,\tau ) = 1,$$

15 $$f_{\xi } \left( {\infty ,\,\tau } \right) \to 0,\,\,f_{\xi \xi } \left( {\infty ,\,\tau } \right) \to 0,\,\,\theta (\infty ,\tau ) \to 0,\phi (\infty ,\tau ) \to 0,\chi (\infty ,\tau ) \to 0,$$

where $K$ (couple stress parameter), $M$ Hartmann number, $S$ ( ratio between oscillating frequency to rate of stretching), $\lambda$ (mixed convection parameter), $E$ (activation energy parameter), $N_{r}$ (buoyancy ratio constant), $\Pr$ (Prandtl number), $R_{b}$ (bioconvection Rayleigh number), $Nb$ (Brownian motion constant), $Rd$ (radiation parameter), $\theta_{w}$ (surface heating parameter) $Nt$ (thermophoresis constant), $\sigma$ (chemical reaction parameter),$Le$ (Lewis number), ${\text{P}} e$ (Peclet number), $\delta$ (temperature difference), $Lb$ (Lewis number) are elaborated as

16 $$\left. \begin{gathered} K = \eta_{0} b/\rho \nu^{2} ,M = \sigma^{ * } B_{0}^{2} /\rho_{f} b,\lambda = \beta \left( {T_{w} - T_{\infty } } \right)\left( {1 - C_{\infty } } \right)/b^{2} x,S = \varpi /b,\Pr = \nu /\alpha_{f} , \hfill \\ Nt = \tau_{1} D_{T} \left( {T_{w} - T_{\infty } } \right)/T_{\infty } \nu ,\sigma = k_{a} /b,Rd = 4\sigma^{ * } T_{\infty }^{3} /3kk^{ * } ,\theta_{w} = T_{w} /T_{\infty } ,E = E_{a} /kT_{\infty } ,Le = \alpha /D_{B} , \hfill \\ Rb = \gamma^{ * } \left( {n_{w} - n_{\infty } } \right)\left( {\rho_{m} - \rho_{f} } \right)/\beta^{ * } \rho_{f} \left( {1 - C_{\infty } } \right)\left( {T_{w} - T_{\infty } } \right),Nb = \tau_{1} D_{B} \left( {C_{f} - C_{\infty } } \right)/\nu ,Le = \nu /D_{B} , \hfill \\ \delta = \left( {T_{w} - T_{\infty } } \right)/T_{\infty } ,Nr = \left( {\rho_{p} - \rho_{f} } \right)\left( {C_{w} - C_{\infty } } \right)/\beta^{ * } \rho_{f} (1 - C_{\infty } )T_{\infty } \beta ,Pe = b_{1} W_{e} /D_{m} ,Lb = \nu /D_{m} \hfill \\ \end{gathered} \right\}$$

It is emphasized that Eq. (11) contains two important parameters namely radiation constant $Rd$ and Prandtl number $\Pr$ and some diverse values may be assigned while performing the graphical analysis.

The executed local Nusselt number $\left( {Nu_{x} } \right),$ local Sherwood number $\left( {Sh_{x} } \right)$ and motile density number $\left( {Nn_{x} } \right)$ are mathematically detected as^12,50,51^:

17 $$Nu_{x} = \frac{{xq_{s} }}{{k\left( {T_{w} - T_{\infty } } \right)}},\,Sh_{x} = \frac{{xj_{s} }}{{D_{B} \left( {C_{w} - C_{\infty } } \right)}},Nn_{x} = \frac{{xj_{n} }}{{D_{n} \left( {n_{w} - n_{\infty } } \right)}},$$

18 $$\frac{{Nu_{x} }}{{\sqrt {{\text{Re}}_{x} } }} = - \left[ {1 + \frac{4}{3}Rd\left( {\theta_{w} - 1} \right)\theta \left( 0 \right) + 1)^{3} } \right]\theta ^{\prime}\left( 0 \right),\theta_{\xi } \left( {0,\tau } \right),\frac{{Sh_{x} }}{{\sqrt {{\text{Re}}_{x} } }} = - \phi_{\xi } \left( {0,\tau } \right),\frac{{Nn_{x} }}{{\sqrt {{\text{Re}}_{x} } }} = - \chi_{\xi } \left( {0,\tau } \right).$$

## Solution methodology

In order to develop analytical expressions for the constituted partial differential Eqs. (10)–(14) with boundary conditions (Eqs. 15, 16), we employ the most interesting technique homotopy analysis technique. This solution technique was basically intended by Liao^47^ and later on many investigations were reported for which the solution procedure was pursued with appliances of homotopic procedure^48–51^. As a first step, we suggest following initial guesses for constituted problem

19 $$f_{0} \left( {\xi ,\tau } \right) = \frac{1}{2}\sin \tau \left[ {3 - 3\exp \left( { - \xi } \right) - \xi \exp \left( { - \xi } \right)} \right],\,\,\,\theta_{0} \left( { - \xi } \right) = \exp \left( { - \xi } \right),\,\varphi_{0} (\xi ) = \exp \left( { - \xi } \right).$$

20 $$\pounds_{f} = \frac{{\partial^{3} }}{{\partial \xi^{3} }} - \frac{\partial }{\partial \xi },\,\pounds_{\theta } = \frac{{\partial^{2} }}{{\partial \xi^{2} }} - 1,\,\pounds_{\phi } = \frac{{\partial^{2} }}{{\partial \xi^{2} }} - 1,\pounds_{\chi } = \frac{{\partial^{2} }}{{\partial \xi^{2} }} - 1,$$

21 $$\pounds_{f} \left[ {\Omega_{1} + \Omega_{2} \exp \left( \xi \right) + \Omega_{3} \exp \left( { - \xi } \right)} \right] = 0,$$

22 $$\pounds_{\theta } \left[ {\Omega_{4} \exp \left( \xi \right) + \Omega_{5} \exp \left( { - \xi } \right)} \right] = 0,$$

23 $$\pounds_{\phi } \left[ {\Omega_{6} \exp \left( \xi \right) + \Omega_{7} \exp \left( { - \xi } \right)} \right] = 0,$$

24 $$\pounds_{\chi } \left[ {\Omega_{8} \exp \left( \xi \right) + \Omega_{9} \exp \left( { - \xi } \right)} \right] = 0,$$

where $a_{i} (i = 1,\,2,...,9)$ are arbitrary constants.

## Convergence of solution

**Figure 2:**
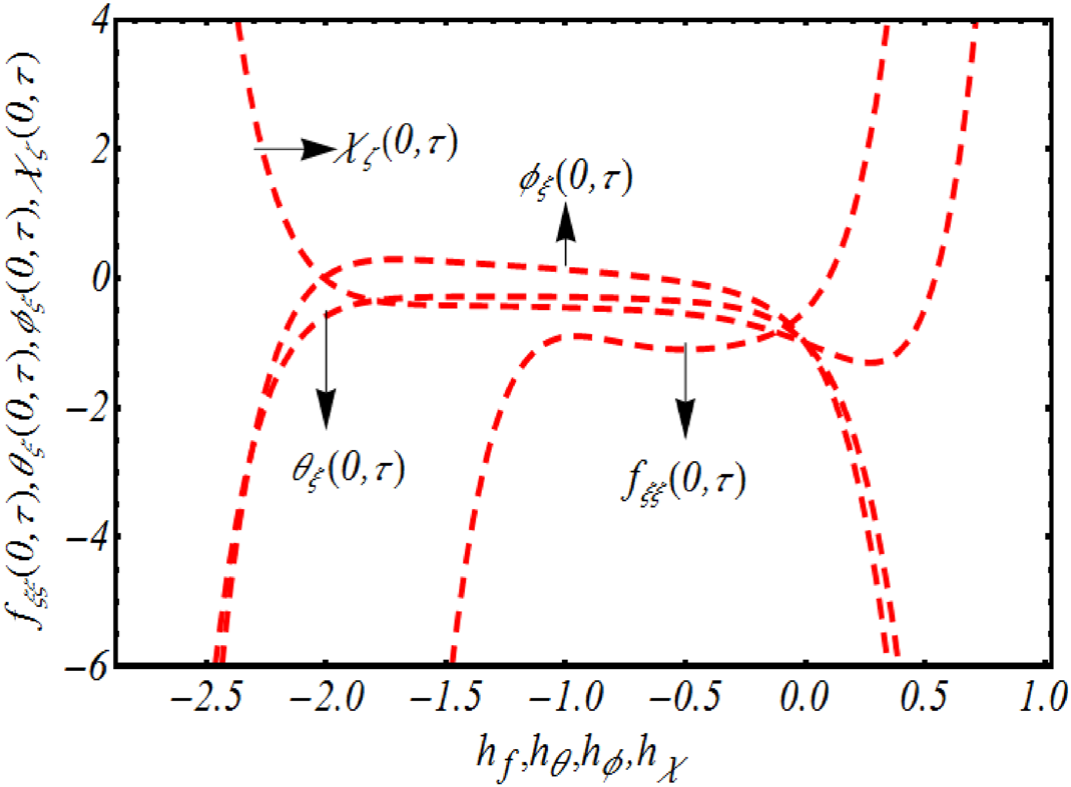
Representation of *h*-curves $f_{\xi \xi } \left( {0,\tau } \right),\,\,\theta_{\xi } \left( {0,\tau } \right),\,\,\phi_{\xi } \left( {0,\tau } \right)$ and $\chi_{\xi } \left( {0,\tau } \right).$

The solution computed via analytical procedure contains most imperative parameters namely auxiliary parameters $$h_{f} ,h_{\theta } ,h_{\phi }$$ and $$h_{\chi }$$ for which specific rang can be defined to better accuracy of solution. For this purpose, we have sketched *h-*curves for velocity, temperature, concentration and gyrotactic microorganism distribution in Fig. 2. While sketching this figure all the flow parameters have assign fixed values like $$K = 0,2,$$ $$\lambda = 0.1,$$ $$M = 0.3,$$$$Rd = 0.1,$$ $$\Pr = 0.4,$$ $$S = 0.2,$$ $$Nb = 0.3,$$ $$Rb = 0.3,$$ $$\theta_{w} = 0.1,$$ $$Nr = 0.2,$$ $$Nt = 0.2,$$ $$E = 0.1,$$ $$Sc = 0.1,$$ $$Pe = 0.3,$$ $$Lb = 0.2,$$ $$\sigma = 0.1$$ and $$\tau = 0.5\pi .$$ It is worth mentioning that better accuracy of numerical simulations is resulted when $$- 1.5 \le h_{f} \le - 0.2,$$ $$- 2.1 \le h_{\theta } \le - 0.1,$$ $$- 2.2 \le h_{\phi } \le - 0.2,$$ $$- 2.1 \le h_{\chi } \le - 0.2.$$

## Validation of solution

The solution procedure and accuracy of obtained results as a limiting case has been verified by comparing current results with already reported investigations in Tables 1 and 2. Table 1 shows the comparison of results with Abbas et al.^43^ and Zheng et al.^44^ against different values of $\tau .$ An excellent accuracy between both numerical computations is noted. The obtained results are further verified with the results claimed by Hayat^49^ and Turkyilmazoglu^49^ in Table 2. Again a favorable accuracy of both results is founded.

**Table 1:** Comparison of $f_{yy} \left( {0,\tau } \right)$ with^12,37^ by keeping $S = 1$, $M = 12,\,\,Nr = 0,\,\,Rb = 0.$

$\tau$	Zheng et al. [^44^]	Abbas et al.^43^	Present results
$\tau = 1.5\pi$	11.678656	11.678656	11.678656
$\tau = 5.5\pi$	11.678706	11.678707	11.678706
$\tau = 9.5\pi$	11.678656	11.678656	11.678656

**Table 2:** Numerical values of $f_{yy} \left( {0,\tau } \right)$ when $\tau = \pi /2,$ $Nr = Rb = S = 0.$

$M$	Turkyilmazoglu [^49^]	Hayat et al. [^52^]	Current results
$0$	− 1.000000	− 1.000000	− 1.000000
$0.5$	− 1.22474487	− 1.224747	− 1.2247470
1	− 1.41421356	− 1.414217	− 1.4142172
1.5	− 1.58113883	− 1.581147	− 1.581147

## Discussion

This section involves the interesting physical significance of flow parameters governed by the dimensionless equations. Since this study is based on theoretical model therefore which need to allocate some arbitrary assigned values to flow involved constants like $$K = 0,2,$$ $$M = 0.3,$$ $$S = 0.2,$$ $$\lambda = 0.1,$$ $$Nr = 0.2,$$ $$Rb = 0.3,$$ $$\Pr = 0.4,Nb = 0.3,$$ $$Nt = 0.2,$$ $$Rd = 0.1,$$ $$\theta_{w} = 0.2,$$ $$Sc = 0.1,$$ $$E = 0.1,$$$$Pe = 0.3,Lb = 0.2,\sigma = 0.1$$ and $$\tau = 0.5\pi .$$

### Velocity distribution

**Figure 3:**
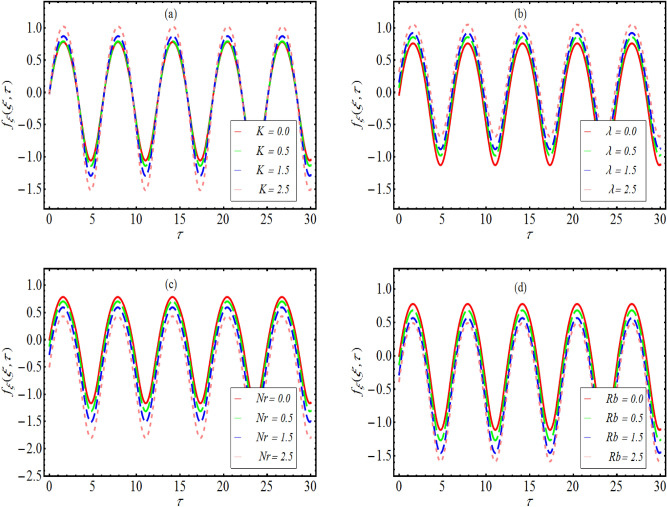
Variation of $f_{\xi }$ with $\tau$ for various values of (**a**) $K$ (**b**) $\lambda$ (**c**) $Nr$ and (**d**) $Rb$.

Figure 3a–d simulates the variation in the velocity distribution $f_{\xi }$ with $\tau$ for various parameters like couple stress $K,$ mixed convection constant $\lambda ,$ buoyancy ratio constant $Nr$ and Rayleigh number $Rb.$ The graphical observations simulated in Fig. 3a convey that the distribution of velocity periodically truncated without any phase shift. Here $K = 0$ corresponds to viscous case for which variation in velocity is quite minimum. However, a dominant velocity distribution is noted for non-Newtonian case due to high viscosity. Physically, the decay in the velocity profile is due to presence of couple stresses. From Fig. 3b, we examined again an increasing velocity distribution by increasing mixed convection constant. The physical interpolation of such trend is justified as mixed convection constant is associated with Grashoff number which yields an impressive enhancement in velocity distribution. The results reported in Fig. 3c deals with the variation of $Nr$ on $f_{\xi } .$ Here, again the velocity distribution shows a sinusoidal behavior as the surface is assumed to be oscillatory. A declined profile of $f_{\xi }$ is observed for $Nr.$ The justified fact for such swelled distribution of velocity is defended as $Nr$ involves buoyancy ratio forces which offer more resistance and fluid particles are not allowed to move freely. Similar observation has been searched out in Fig. 3d.

### Temperature distribution

**Figure 4:**
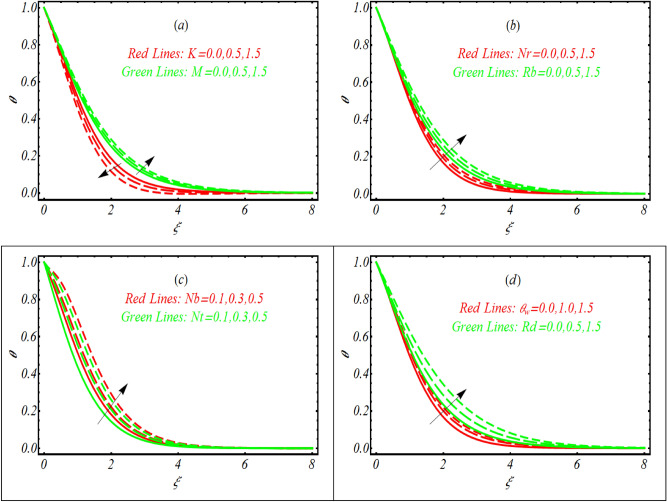
Variation of $\theta$ for various values of (**a**) $K$ and $M$ (**b**) $Nr$ and $Rb$ (**c**) $Nt$ and $Nb.$(**d**) $Rd$ and $\theta_{w} .$

Now let us check the alteration in nanofluid temperature $\theta$ and development in associated thermal boundary layer due to diverse values of couple stress parameter $K,$ thermophoresis parameter $Nt,$ Hartmann number $M,$ Rayleigh number $Rb,$ buoyancy ratio constant $Nr,$ and Brownian motion constant $Nb,$ Fig. 4a–d is prepared. Figure 4a exposed the joint features of couple stress parameter $K$ and Hartmann number $M$ on $\theta .$ The temperature distribution get maximum value with variation of $M.$ The physical explanation due to such enrich temperature distribution may justified as interaction of magnetic field results Lorentz force which increases the nanoparticles temperature effectively. However, opposite trend has been reported in case of $K.$ A declining distribution of $\theta$ is figured out with $K$ due to presence of couple stresses. From Fig. 4b, an improved and more thermal boundary layer is found due to variation of $Nr$ and $Rb.$ The variation in both physical parameters is attributed to the buoyancy forces which help to enhanced the nanoparticles temperature. Further, the thickness of thermal boundary layer is quite stable. The truncation in $\theta$ due to $Nt$ and $Nb$ is inspected in Fig. 4c. Thermophoresis phenomenon is based on the migration of accelerated particles towards cool region. The fluctuation in temperature is resulted from migration of fluid particles from hot region. Similarly temperature distribution increases due to increment in $Nb.$ Brownian movement is random molecular movement of fluid particles due to which the temperature distribution reached at maximum level. Figure 4d capture the graphical results for radiation parameter $Rd$ and surface heating constant $\theta_{w}$ on $\theta .$ The curve of temperature $\theta$ get arises with increment of both $Rd$ and $\theta_{w} .$ Here $\theta_{w} = 1$ corresponds to the linear radiation while $\theta_{w} = 1.0,1.5$ are associated with the nonlinear radiative case. With utilization of nonlinear thermal radiation, a more complicated and nonlinear heat equation is resulted. Unlike linear thermal radiation, the change in temperature is more progressive by using nonlinear thermal radiation consequences.

### Concentration distribution

**Figure 5:**
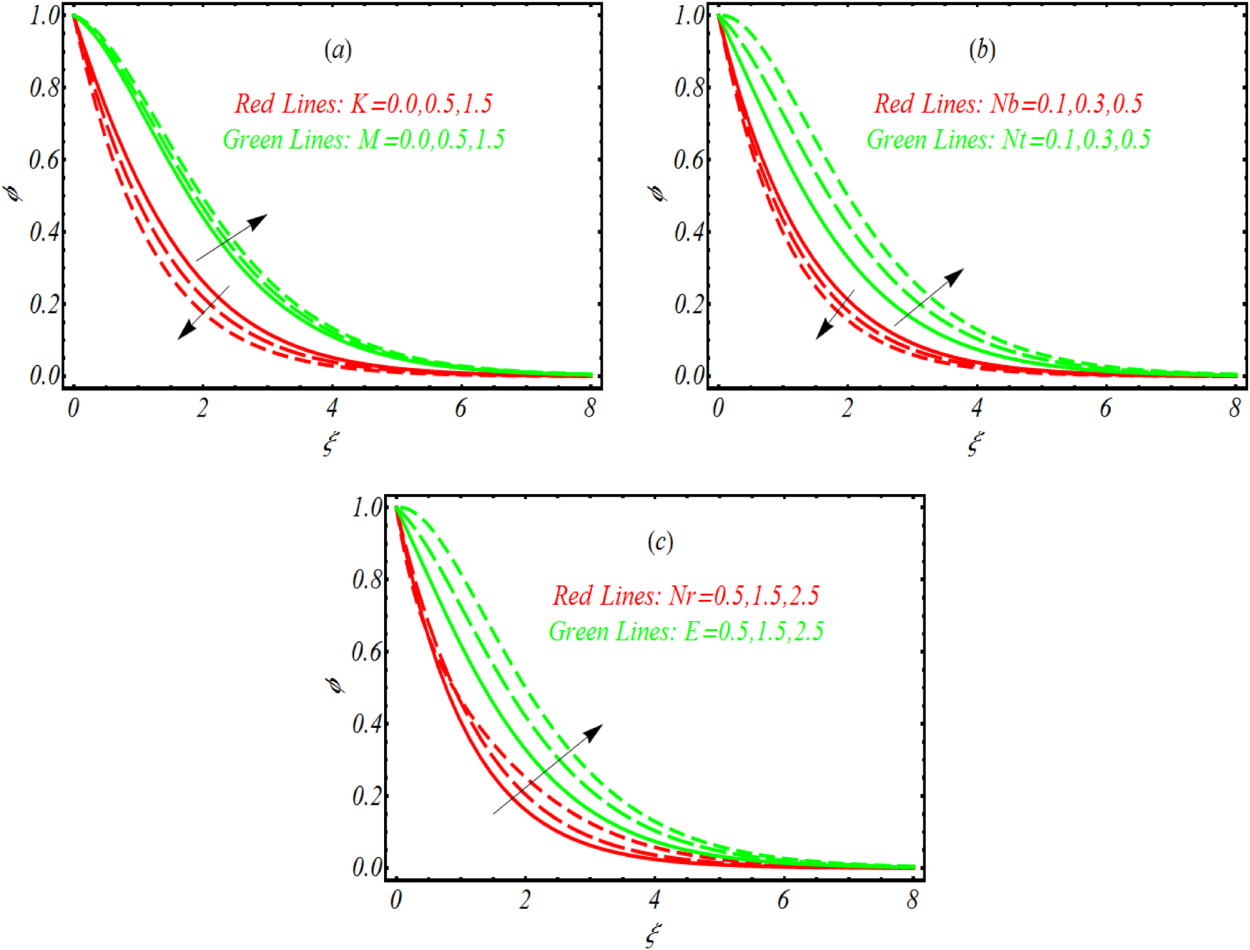
Variation of $\phi$ for various values of (**a**) $K$ and $M$ (**b**) $Nt$ and $Nb$ (**c**) $Nr$ and $E.$

Figure 5a–c characterizes the physical consequences of couple stress parameter $K,$ Hartmann number $M,$ thermophoresis parameter $Nt$ and Brownian motion constant $Nb,$ buoyancy ratio constant $Nr$ and activation energy parameter $E$ on concentration $\phi .$ The analysis worked out in Fig. 5a reports that a lower change in $\phi$ is observed as $K$ assign maximum values. However, concentration distribution enhanced due to interaction of magnetic field. The utilization of magnetic force results Lorentz force which increases the nanofluid concentration effectively. The graphical observations in order to notice the change in concentration profile due to variation of thermophoresis parameter $Nt$ and Brownian motion constant $Nb$ is determined in Fig. 5b. An enrich concentration distribution is noted when $Nt$ assigned maximum values. On contrary the concentration distribution declined with $Nb.$ The decrement in concentration profile due to Brownian motion $Nb$ is justified as $Nb$ attain reverse relation which dimensionless concentration Eq. (12). From Fig. 5c it is visualized that presence of activation energy $E$ and buoyancy ratio constant $Nr$ increases the concentration distribution efficiently. The roll of activation energy in various chemical processes is important as it enhanced the reaction rate. Similarly, enrollment of buoyancy forces also play sufficient role to improve the nanoparticles concentration.

### Motile microorganism distribution

**Figure 6:**
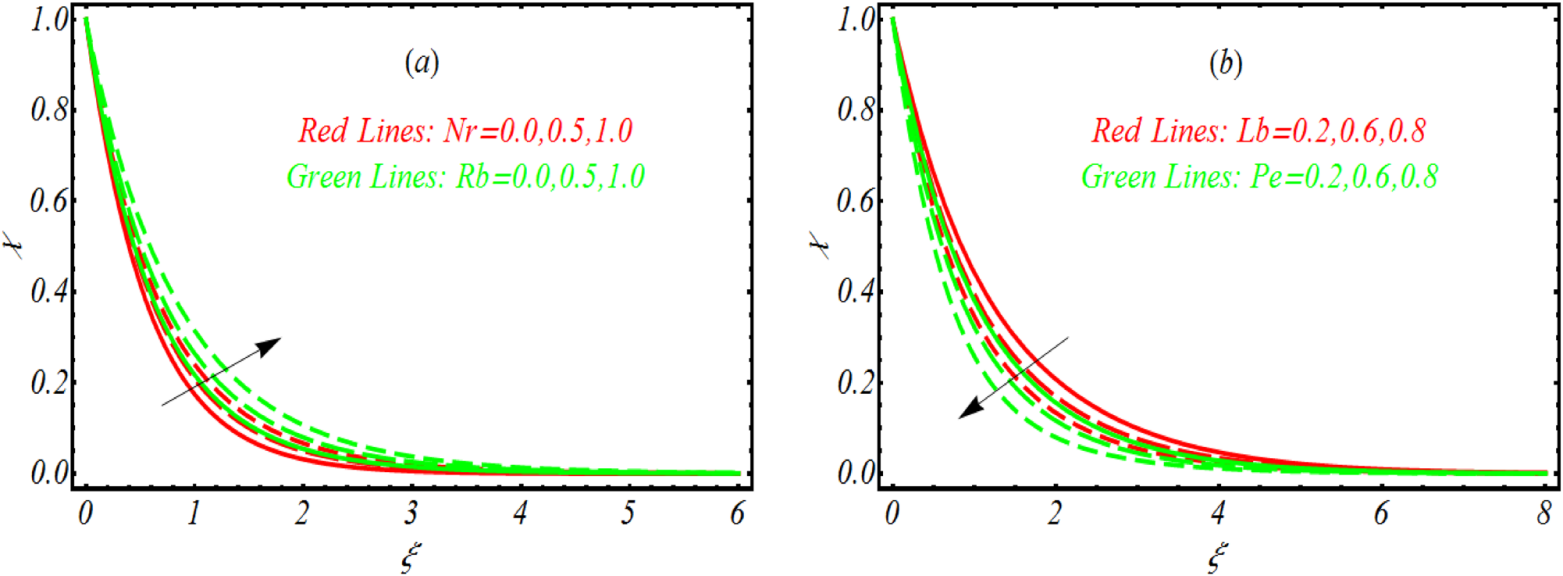
Variation of $\chi$ for various values of (**a**) $Nr$ and $Rb$ (**b**) $Lb$ and $Pe.$

The graphical analysis for microorganism distribution $\chi$ for various values of $Nr,$ Rayleigh number $Rb,$ buoyancy parameter, bioconvection Lewis number $Lb$ and Peclet number $Pe$ is presented in Fig. 6a,b. Figure 6a presents variation in $\chi$ for $Nr$ and $Rb.$ A remarkable enhancement in $\chi$ is observed for both parameters which is referred to buoyancy forces. The graphical results for $Lb$ and $Pe$ are notified in Fig. 6b. The microorganism distribution declined slightly for both flow parameters. In fact, $Pe$ allows reverse relations with motile diffusivity due to which microorganism becomes weaker.

### Skin friction coefficient

**Figure 7:**
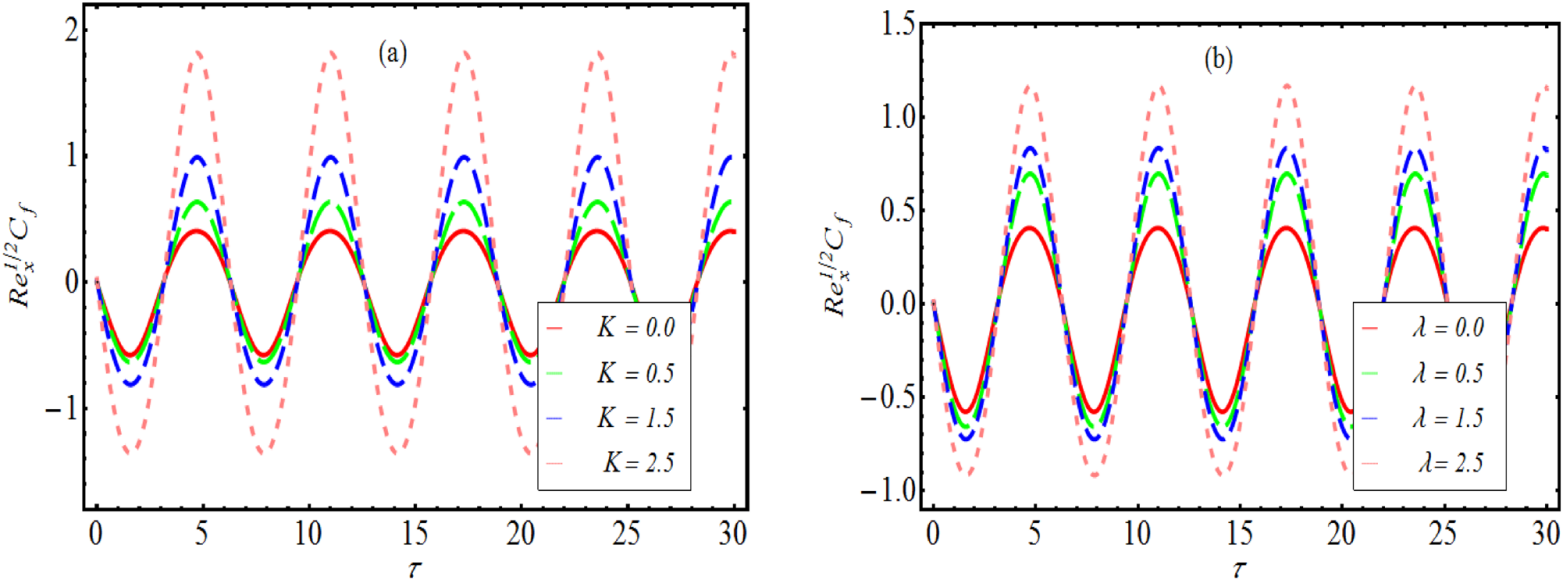
Variation of ${\text{Re}}_{x}^{1/2} C_{f}$ for various values of (**a**) $K$ and (**b**) $\lambda .$

In order to examine the variation in most interesting physical quantity namely wall shear force for assigned values of $K,$ and $\lambda ,$ Fig. 7a,b is utilized. From both figure, it is noted that skin friction coefficient oscillates periodically and the magnitude of oscillation increases with both $K$ and $\lambda .$ The periodical fluctuation in wall shear force is due to fact that considered stretched configuration is assumed to be periodically accelerated. However, magnitude of oscillation for $K$ is relatively larger as compared to $\lambda .$

### Physical quantities

**Figure 8:**
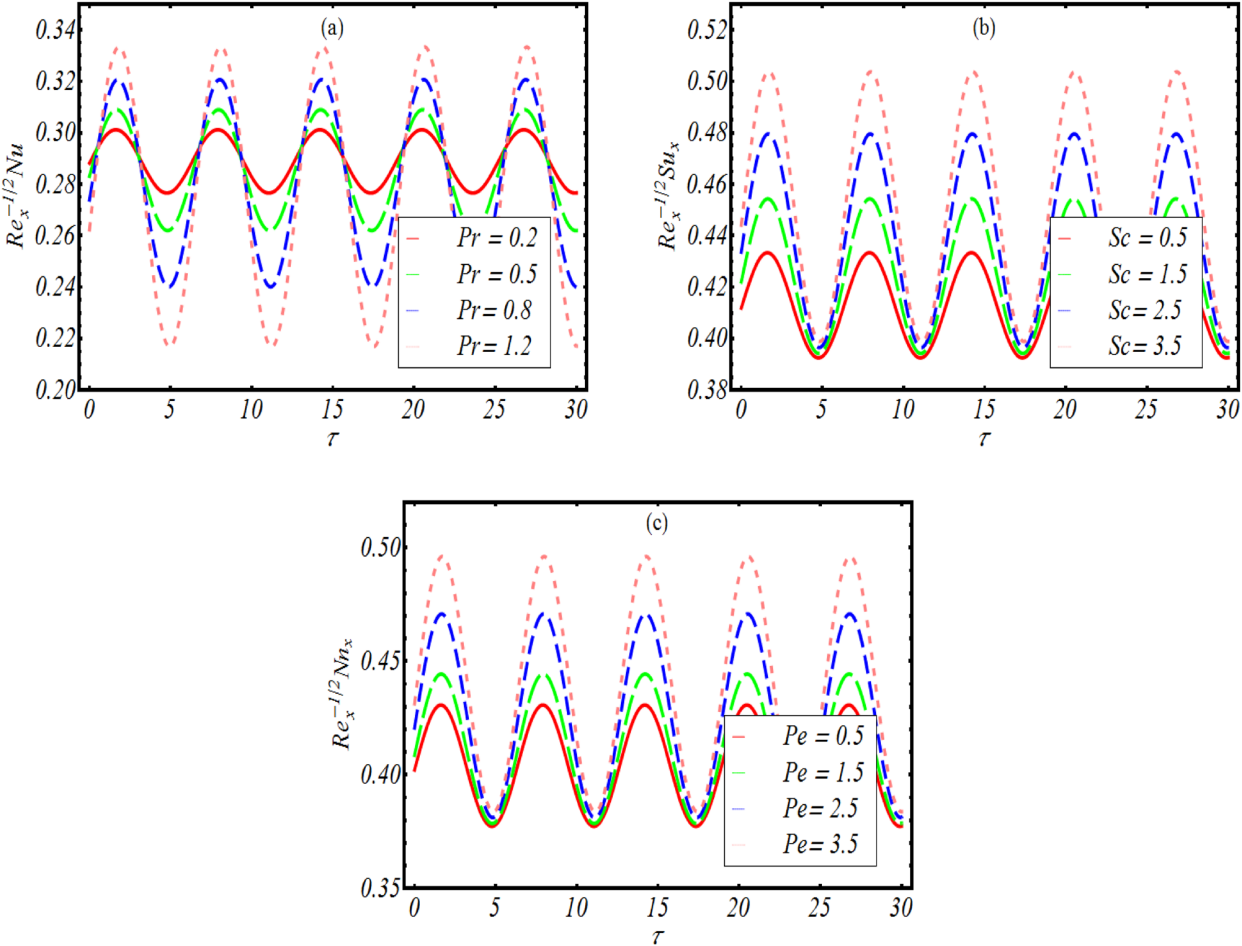
(**a**) Variation in ${\text{Re}}_{x}^{1/2} Nu_{x}$ for $\Pr ,$ (**b**) variation in ${\text{Re}}_{x}^{1/2} Sh_{x}$ for $Sc$ and (**c**) variation in ${\text{Re}}_{x}^{1/2} Nn_{x}$ for $Pe.$

Figure 8a represents the change in local Nusselt number ${\text{Re}}_{x}^{1/2} Nu_{x}$ against time $\tau$ due to change in $\Pr .$ The local Nusselt number vacillate periodically with $\tau$ and rate of oscillation get maximum amplitude for privilege values of $\Pr .$ Figure 8b reports the influence of $Sc$ on local Sherwood number ${\text{Re}}_{x}^{1/2} Sh_{x}$ which is varies against $\tau .$ An increasing periodically variation of local Sherwood number is noticed with $Sc.$ Figure 8c signifies that motile density number ${\text{Re}}_{x}^{1/2} Nn_{x}$ also shows increasing trend with variation of $Pe.$ The numerical illustration of ${\text{Re}}_{x}^{1/2} Nu_{x}$, ${\text{Re}}_{x}^{1/2} Sh_{x}$ and ${\text{Re}}_{x}^{1/2} Nn_{x}$ against different physical parameters are shown in Table 3. It is noted that all these quantities increases for couple stress parameter $K$ while these quantities decreases for Hartmann number $M$ and effective Prandtl number $\Pr$ and thermophoresis parameter $Nt.$

**Table 3:** Numerical iteration for $- \theta_{\xi } \left( {0,\tau } \right),\,\, - \phi_{\xi } \left( {0,\tau } \right)$ and $- \chi_{\xi } \left( {0,\tau } \right)$ for various parameters at $\tau = 0.5\pi .$

$K$	$M$	$\lambda$	$Nr$	$Rb$	$Nb$	$Nt$	$\Pr$	$Nu_{x} /\sqrt {{\text{Re}}_{x} }$	$Sh_{x} /\sqrt {{\text{Re}}_{x} }$	$Nn_{x} /\sqrt {{\text{Re}}_{x} }$
0.1	0.2	0.3	0.2	0.1	0.3	0.4	0.2	0.54668	0.48546	0.588654
0.3								0.58397	0.49666	0.60325
0.5								0.62168	0.53154	0.64287
0.2	0.3							0.53530	0.50754	0.57908
	0.6							0.52456	0.47650	0.56432
	0.8							0.50121	0.45687	0.54609
		0.2						0.53378	0.46554	0.56986
		0.5						0.55628	0.49806	0.59053
		0.7						0.59390	0.51325	0.63576
			0.4					0.54345	0.48975	0.56794
			0.6					0.53155	0.46665	0.55021
			0.8					0.51530	0.43598	0.535743
				0.3				0.53578	0.47435	0.57520
				0.5				0.51990	0.45675	0.53065
				0.7				0.46520	0.41577	0.515896
					0.1			0.52520	0.48562	0.56741
					0.4			0.50825	0.51887	0.52632
					0.6			0.46675	0.54358	0.50503
						0.3		0.54759	0.49675	0.57764
						0.5		0.51842	0.47512	0.54890
						0.6		0.86590	0.44389	0.52348
							0.1	0.53583	0.48678	0.56776
							0.5	0.62792	0.51489	0.59092
							0.7	0.69823	0.54730	0.63702

## Conclusions

A theoretical model based on the flow couple stress nanofluid containing gyrotactic microorganism over an accelerated stretched surface has been evaluated analytically. The interesting features of activation are also entertained in the concentration equation. The radiation effects are simplified via one parametric approach by utilizing the Prandtl number and radiation constant. Main results reported from current contribution are listed as:

- The velocity distribution periodically accelerated and magnitude of oscillation increases with couple stress.
- The temperature of nanoparticles reduces with couple stress parameter while it improves with Brownian constant and thermophoresis parameter.
- The activation energy parameter increases the concentration distribution while Brownian motion constant and couple stress parameters declined the concentration profile.
- The gyrotactic microorganism distribution becomes weaker for Peclet number and bioconvection Lewis number in contrast to buoyancy ratio constant and Rayleigh number.
- The skin friction coefficient increases with couple stress parameter and mixed convection constant.
- The reported scientific contribution may helpful for improvement of extrusion processes, enhancement of heat transfer, biotechnology and bio-fuels.
- The simulations presented in the work can be further extended for three-dimensional flows in presence of various flow features like nanofluids, activation energy, Joule heating, thermal radiation and entropy generation features. Moreover, various numerical schemes can also be employed for such formulated accelerated surfaces problems.

## Appendix

### Governing equation for couple stress fluid

The equations of motion governing the flow of a couple stress fluid in presence of magnetic field and porous media are given by^24^:

25 $$\rho \left( {{\mathbf{V}}.{\mathbf{\nabla }}} \right){\mathbf{V}} = - \nabla p + \left( {\lambda + \mu } \right)\nabla^{2} {\mathbf{.V + }}\eta \Delta \nabla \nabla {\mathbf{.V}} - \eta .\Delta^{2} {\mathbf{V + }}\mu \nabla {\mathbf{V + }}\sigma \left( {{\mathbf{V}} \times {\mathbf{\rm B}}} \right) \times {\mathbf{{\rm B} + }}\rho f + \frac{1}{2}\nabla \times \left( {\rho {\mathbf{\rm I}}} \right),$$

In above equations ${\mathbf{V}}$ is the velocity vector, ${\mathbf{J}}$ is the current density, ${\mathbf{B}}$ is the magnetic flux vector , $\rho$ is the density of the fluid, $d/dt$ represents the material derivative, $\Delta$ is Laplacian operator, $p$ is the pressure, $\lambda$ and $\mu$ are the viscosity coefficients, $\eta$ is couple stresscoefficients. ${\mathbf{f}}$ is a body force and ${\mathbf{I}}$ is a body couple moment. For couple-stress fluid, shear stress tensor is not symmetric. The mathematical expressions for force stress tensor $\tau$ and the couple-stress tensor ${\mathbf{\rm M}}_{{\mathbf{1}}}$ that arises in the couple-stress fluids theory are expressed by

26 $$\begin{gathered} \tau = \left( { - p + \lambda \nabla {\mathbf{V}}} \right){\mathbf{I}} + \mu \left( {\nabla {\mathbf{V + }}\left( {\nabla {\mathbf{V}}} \right)^{T} } \right) + \frac{1}{2}{\mathbf{I}} + \left( {\nabla {\mathbf{M}}_{{\mathbf{1}}} {\mathbf{ + }}\rho {\mathbf{\rm I}}} \right), \hfill \\ {\mathbf{M}}_{{\mathbf{1}}} {\mathbf{ = }}m{\mathbf{I + }}2\eta \left( {\nabla \left( {\nabla \times {\mathbf{V}}} \right)} \right) + 2\eta^{\prime}\left( {\nabla \left( {\nabla \times {\mathbf{V}}} \right)} \right)^{T} , \hfill \\ \end{gathered}$$

where $\eta ^{\prime}$ are couple stress coefficients. Further, the materials constants $\lambda$ and $\mu ,$ $\eta$ and $\eta ^{\prime}$ satisfy the following inequalities:

27 $$\left. \begin{gathered} \mu \ge 0, \hfill \\ 3\lambda + 2\mu \ge 0, \hfill \\ \eta \ge 0, \hfill \\ \eta \ge \eta ^{\prime}, \hfill \\ \end{gathered} \right\}$$

In the absence of body force and body couple moment (Eq. 25) reduce to

28 $$\rho \left( {{\mathbf{V}}.{\mathbf{\nabla }}} \right){\mathbf{V}} = - \nabla p + \mu \nabla^{2} {\mathbf{V}} - \eta \nabla^{4} {\mathbf{V + }}\sigma \left( {{\mathbf{V}} \times {\mathbf{\rm B}}} \right).$$

Further, we have used the generalized Ohm’s law to write

29 $${\mathbf{J = }}\sigma_{e} \left( {{\mathbf{V}} \times {\mathbf{B}}} \right),$$

in which $\sigma_{e}$ is the electrical conductivity.

For flow under consideration, the appropriate velocity field is

30 $${\mathbf{V}} = \left[ {u\left( {x,y,t} \right),v\left( {x,y,t} \right),0} \right]$$

By employing boundary layer approximations, above set of equations yield following governing equation for couple stress fluid

31 $$\frac{\partial u}{{\partial t}} + u\frac{\partial u}{{\partial x}} + v\frac{\partial u}{{\partial y}} = \nu \frac{{\partial^{2} u}}{{\partial \overline{y}^{2} }} - \frac{{\eta_{0} }}{{\rho_{f} }}\frac{{\partial^{4} u}}{{\partial y^{4} }} - \frac{{\sigma_{e} B_{0}^{2} }}{{\rho_{f} }}u,$$
